# Attention-Fusion-Based Two-Stream Vision Transformer for Heart Sound Classification

**DOI:** 10.3390/bioengineering12101033

**Published:** 2025-09-26

**Authors:** Kalpeshkumar Ranipa, Wei-Ping Zhu, M. N. S. Swamy

**Affiliations:** Department of Electrical and Computer Engineering, Concordia University, Montreal, QC H3G 1M8, Canada; weiping@ece.concordia.ca (W.-P.Z.); swamy@ece.concordia.ca (M.N.S.S.)

**Keywords:** attention fusion, vision transformer, deep learning, heart sound classification

## Abstract

Vision Transformers (ViTs), inspired by their success in natural language processing, have recently gained attention for heart sound classification (HSC). However, most of the existing studies on HSC rely on single-stream architectures, overlooking the advantages of multi-resolution features. While multi-stream architectures employing early or late fusion strategies have been proposed, they often fall short of effectively capturing cross-modal feature interactions. Additionally, conventional fusion methods, such as concatenation, averaging, or max pooling, frequently result in information loss. To address these limitations, this paper presents a novel attention fusion-based two-stream Vision Transformer (AFTViT) architecture for HSC that leverages two-dimensional mel-cepstral domain features. The proposed method employs a ViT-based encoder to capture long-range dependencies and diverse contextual information at multiple scales. A novel attention block is then used to integrate cross-context features at the feature level, enhancing the overall feature representation. Experiments conducted on the PhysioNet2016 and PhysioNet2022 datasets demonstrate that the AFTViT outperforms state-of-the-art CNN-based methods in terms of accuracy. These results highlight the potential of the AFTViT framework for early diagnosis of cardiovascular diseases, offering a valuable tool for cardiologists and researchers in developing advanced HSC techniques.

## 1. Introduction

Cardiovascular diseases (CVDs) are the predominant global factor contributing to mortality, resulting in the loss of over 17 million lives annually and posing a severe threat to human health in daily life [1]. Early screening of CVDs is essential in reducing mortality, especially in small primary care clinics. Traditionally, examining heart sounds has been a vital method for identifying CVDs, as it offers important insights into cardiovascular disorders. However, identifying cardiac abnormalities through auscultation can be notably challenging, particularly for clinicians with limited expertise. Experienced cardiologists can accurately diagnose heart disease with a precision of 73% to 80%, whereas inexperienced new physicians or trainees achieve a lower accuracy rate of 20% to 40% [2,3]. Hence, the development of automated approaches by applying signal processing techniques for HSC holds significant promise for enhancing heart condition diagnosis and treatment in these regions.

Recent advancements in AI technologies, such as machine learning and deep-learning-based algorithms, are revolutionizing cardiovascular care. Existing algorithms for HSC can be divided into two major groups, namely, single-stream models and multi-stream models. A single-stream framework utilizes only one classifier for the classification task. It takes the raw heart sound signal or a single extracted feature as input. In [4], traditional single-stream machine learning approaches, namely, decision tree (DT), naive Bayes (NB), support vector machine (SVM), K-nearest neighbor (KNN), and the ensemble method (EM), are employed for different domain features, such as time, frequency, high-order statistics, energy, and mel-frequency cepstral coefficients (MFCCs). However, these methods often struggle with issues such as noise sensitivity, excessive dependence feature extraction and limited robustness. In contrast, deep learning methods offer significant advantages, as they can automatically identify relevant features and uncover hidden patterns within complex heart sound signals. Deep learning architectures, such as convolutional neural networks (CNNs), recurrent neural networks (RNNs), and attention-based techniques, have shown exceptional proficiency in analyzing complex signals. In [5], the authors used a one-dimensional convolutional neural network (1D-CNN) and a feed-forward neural network (FNN) for raw signal input. In [6], the authors trained convolutional neural networks (CNNs) separately on the melspectrum and log-melspectrum, and showed that the log-melspectrum can enhance the performance of CNNs for HSC. RNNs have been widely utilized in natural language processing (NLP) applications, including tasks like speech recognition and language translation. RNNs are prone to the vanishing gradient problem, limiting their effectiveness in modeling long-range dependencies. To overcome this, the long short-term memory (LSTM) variant was developed, incorporating gating mechanisms to control information flow and alleviate gradient issues. In [7], the authors implemented two models, namely CNN and LSTM (a variant of RNN), using log-mel spectrogram features extracted from heart sound signals. Their results demonstrated that the CNN model achieved higher accuracy than the LSTM model for multi-class HSC. Moreover, to enhance classification accuracy, the authors of [8] introduced a CNN-BiLSTM model, a cascading network that integrates a convolutional neural network (CNN) with a bi-directional long short-term memory (Bi-LSTM) network, utilizing features such as wavelet coefficients and z-score. In [9], the authors employed spatial and channel attention mechanisms to enhance the feature representations extracted by the CNN, effectively addressing the issue of redundant or identical feature maps. Transfer learning is a widely used approach in deep learning, especially in CNN models, as it accelerates the learning process and enhances the model’s ability to generalize. In [10], the authors utilized transfer learning models, namely AlexNet, VGG16, InceptionV3, and ResNet50, by converting heart sound signals into chaogram image features. The authors of [11] employed three transfer learning models, namely VGG16, VGG19, and ResNet50, to evaluate MFCC’s and Spectrogram’s features and compared their performances. In [12], transfer learning models such as VGG16, ResNet50, and InceptionV3 are utilized to extract features, including log-mel spectrograms, waveforms, and log-power spectrograms, from heart sound signals. The performances of several architectures, including LeNet5, AlexNet, ResNet50, VGG16, and GoogLeNet, are compared in [13] by extracting temporal features and time-frequency features. Similarly, researchers utilized pre-trained ResNet50 in [14] and wav2vec in [15] with raw signal inputs. However, it is worth noting that these transfer learning models necessitate a large number of training parameters, rendering them computationally intensive.

The above-mentioned single-stream methods can produce biased results if less informative features are selected for the classification task. Unlike single-stream methods, multi-stream architectures use a variety of complementary features to improve classification accuracy and represent complex scenarios. Multi-stream methods can be broadly characterized by the stage where the information from the inputs is fused: early, late, and feature-level fusion. The initial category, early fusion, involves merging feature vectors at the input stage without weighting, followed by classification. In [16], MFCCs’ features are fused with 1D adaptive local ternary features to improve robustness, and these combined features are analyzed separately using decision tree, KNN, and SVM classifiers. Similarly, in [17], MFCCs and discrete wavelet transform features are initially combined and then independently classified using SVM, KNN, and deep neural network classifiers. In [18], MFCCs and 1D local ternary patterns are integrated and classified using an SVM. In [19], wavelet synchrosqueezing transform is used to extract magnitude and phase features, which are then fused and classified with a random forest (RF) classifier. The authors of [20] employed an early fusion strategy to combine two features, namely Fbank and MFCCs, which were subsequently classified using the ResNet model. Despite utilizing all available information, early fusion does not exploit the interdependencies among the different domain features. Additionally, the feature vector formed at the input stage is often large and prone to redundancy. On the other hand, late fusion, the second type of fusion method, is a broadly adopted approach. This architecture involves multiple branches, each containing feature extractors and classifiers. The outputs from each of these branches are then used to make the final decision based on concatenation, logical AND, product rule, sum rule, or majority voting techniques. In [21], a three-branch network utilizing ResNet was employed, processing log-mel spectrogram input features with late fusion applied for classification. The authors of [22] utilized a late fusion framework in which four CNNs were employed, each taking MFCCs and their delta features as input. The final classification decision is calculated through a majority voting mechanism. However, the limitation of this method is that it relies on a manual segmentation step, such as the location of systole, diastole, and s1 and s2 features. One significant limitation of the late fusion technique is its requirement for N classifiers when dealing with N different modalities, which leads to increased computational complexity. Furthermore, this approach lacks the ability to capture the interdependencies among feature maps, a crucial aspect for enhancing overall performance.

Feature-level fusion represents the third category of fusion algorithms designed to mitigate the drawbacks of the early and late fusion approaches. This approach leverages the intrinsic relationships among various feature maps, with each domain contributing distinct feature maps. The authors of [23] have extracted time-domain features using three parallel branches of CNN-based feature extractors. The resulting feature maps are further enhanced through channel-wise and temporal attention mechanisms, and then they are classified using a softmax classifier to predict the respective class. The authors of [24] employed a two-stream architecture with raw signal inputs, incorporating pre-trained audio neural networks (PANNs) and feature-level fusion. However, a significant limitation of these methods is its reliance on raw signals instead of domain-specific features. The use of raw signals fails to capture the more meaningful representations that domain-specific features provide. Incorporating these features is essential for improving the model’s overall performance. In contrast, [25] explores the analysis of multidomain features. The authors employ three parallel CNN branches, each processing distinct feature types, namely, MFCCs, mel-domain, and general-domain features, extracted from raw heart sounds. The resulting feature maps are then concatenated at the feature level and fed into a fully connected layer, followed by a softmax classifier. Two parallel CNN branches are fused at the feature level in [26], each processing input high-order spectral features and short-time Fourier transform (STFT) features, respectively. In [27], a feature-level fusion method is implemented, in which second-order spectral analysis features are separately fed into a parallel network composed of two CNN branches and one transformer branch. The majority of the above-mentioned multi-stream methods employ addition, pooling, averaging, or concatenation operations for fusing the extracted features. Although these fusion strategies improve classification results compared to single-stream models, they have limitations, such as failing to consider interactions between multiple features and treating all features equally without leveraging the unique contribution of any specific features. Consequently, this type of fusion does not explore the relationships between features and cannot adjust the importance of each feature during processing.

To address this, attention-based methods have been proposed, which dynamically focus on the most informative feature. The attention module enables the model to prioritize various aspects of the input, enhancing its ability to recognize significant patterns within the data. The transformer architecture, originally developed for natural language processing (NLP) [28], achieves outstanding results across various language tasks, particularly with models like bidirectional encoder representations from transformers (BERTs) [29] and generative pre-trained transformers (GPTs) [30]. Transformers utilize a self-attention mechanism that captures relationships within a sequence, offering the ability to process entire sequences, thereby learning long-range dependencies, enabling efficient parallelization, and scaling to complex models and large datasets. The success of transformer networks in NLP has sparked significant interest within the computer vision research community. In light of the rapid development of vision architectures, several recent models have significantly extended large-kernel designs using ConvNets and hybrid frameworks. In particular, the OverLoCK model [31] adopts a hierarchical approach characterized by an “overview-first, look-closely-next” mechanism, employing large receptive fields in conjunction with context mixing. Another example is the hybrid UniRepLKNet [32], which integrates large-kernel convolutions with transformer-based representations to jointly capture spatial and channel-wise attention. While these models improve feature reuse through block-level modifications, they also introduce increased computational costs and memory requirements, particularly in deep network configurations. In parallel with the progress in convolutional vision architectures, state-space models have emerged as an alternative choice. Vision Mamba models, such as VSSD [33], MSVMamba [34], and SparX-Mamba [35], are proposed to support scalable context aggregation and multiscale representation learning. However, these models often lack domain-specific inductive biases, which are particularly important for medical applications. It is also worth noting that these models are developed for image processing and object-level recognition tasks. On the other hand, heart sound classification remains a challenging task due to limited data availability and high inter-class similarity among disease categories.

Recently, transformers have been applied to HSC, with most recent studies building on the classical ViT models. In [36], the ViT architecture is utilized to extract time-frequency features, which are then classified using a softmax classifier to determine the target class. Similarly, wavelet features are used as input to a ViT transformer model in [37]. The resulting feature maps are then classified using fully connected layers followed by a softmax function for final classification. In [38], the authors utilized a cascade network called Conv-DeiT, where a CNN branch processes MFCC features, which are then further refined by a data-efficient image transformer (DeiT) model. Although DeiT models, especially when pre-trained on image datasets, are effective, they are computationally intensive. This high computational demand may limit their practicality in some applications. Combining features can enhance classification results, but selecting and integrating multiple features remains a significant challenge. To address the inefficiencies of single-stream Vision Transformer models and the lack of closer feature fusion interactions in two-stream models, we propose a novel model called the attention fusion-based two-stream Vision Transformer (AFTViT) for HSC. Specifically, we introduce a dual-branch transformer that merges two patches of different sizes to enhance HSC accuracy. Our primary focus in this study is to develop an appropriate feature fusion block tailored for Vision Transformers, an area that has been relatively underexplored thus far. In particular, we introduce an efficient attention block where each transformer branch’s output is processed through attention mechanisms to produce enriched feature maps. The proposed framework is designed to extract and integrate complementary and discriminative features with reduced dimensionality. The proposed work consists of the following key contributions:

- We propose an efficient AFTViT architecture that effectively learns signal representations from features at different resolutions. By leveraging multi-resolution features, the proposed approach addresses the limitations of single-scale representations, thereby improving the accuracy of HSC.
- A distinctive attention fusion block (AFB) is introduced to integrate the feature representations derived from the small-patch ViT branch and the large-patch ViT branch. Additionally, we evaluate the importance of the proposed AFB by evaluating it against traditional techniques, demonstrating its superiority in achieving robust classification performance.
- We assess the performance of our proposed method on two widely used datasets to validate the effectiveness of the dual-branch approach. The results reveal that the proposed AFTViT outperforms the baseline CNN models and state-of-the-art approaches while maintaining significantly lower parameters because of its efficient design.

The organization of the paper is as follows: Section 2 introduces the proposed AFTViT model, detailing its architecture and components. Section 3 outlines the experimental setup and conducts a comprehensive study of the performances of the proposed method with comparison to existing schemes. Finally, Section 4 concludes the proposed work.

## 2. Proposed AFTViT Architecture

**Figure 1:**
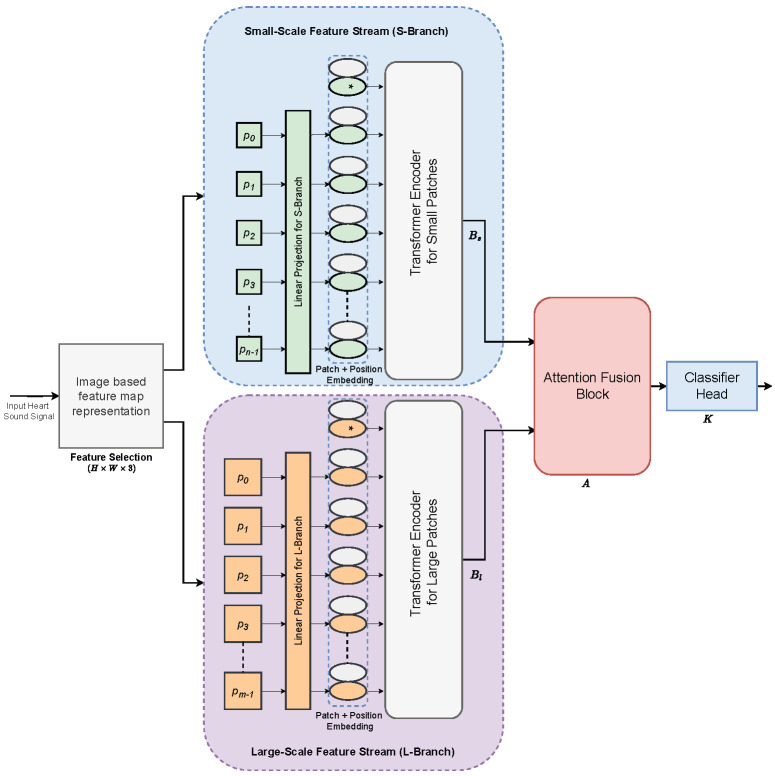
Overall schematic representation of the proposed AFTViT architecture. The ‘⁎’ symbol indicates the learnable [class] token used for classification.

The proposed AFTViT network is shown in Figure 1. To more effectively leverage the discriminative information contained in heart sounds, we employ an image-based feature representation by integrating MFCC and its delta features. The proposed composite model integrates two transformer branches, namely, the small-scale feature stream (S-branch) and the large-scale feature stream (L-branch), which, respectively, extract a complementary set of features for HSC. Subsequently, a fusion block is utilized for integrating features from both streams. In addition, an attention mechanism is employed in the fusion block to emphasize the valuable information of transformer block feature maps while suppressing the remaining details. In the following subsection, the detailed architecture and its components are discussed.

### 2.1. Feature Selection

**Figure 2:**
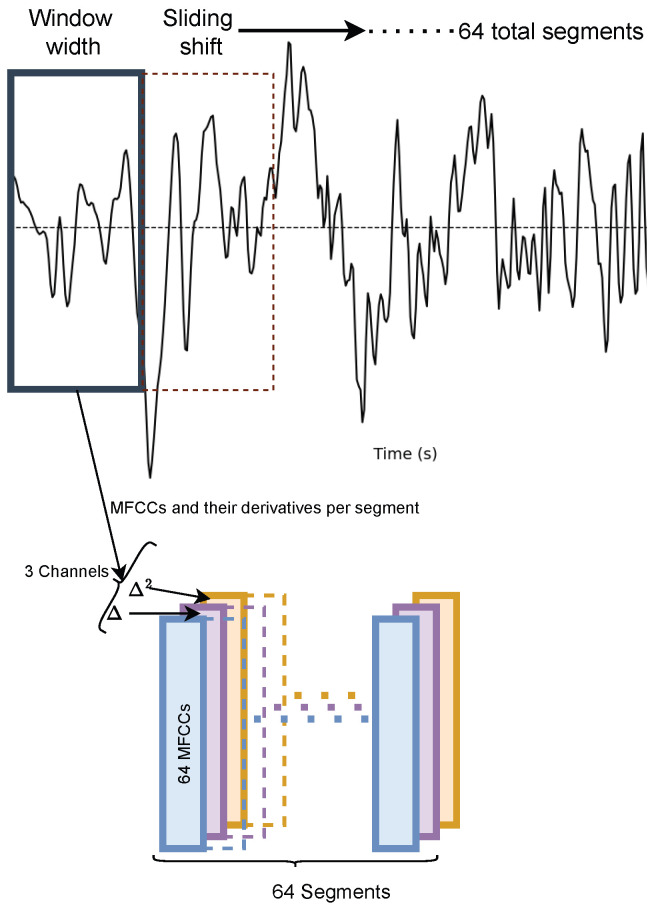
Image-based representation of heart sound using MFCCs and delta features.

Selecting appropriate features is crucial for the implementation of ViT. In our architecture, the Vision Transformer operates on three-dimensional inputs, as shown in Figure 2. To align with this requirement, we leverage MFCCs, delta, and double-delta features as the three-dimensional representation, effectively simulating the expected input structure. Deltas are known as differential and acceleration coefficients, and they are computed from the difference between the current coefficients and the previous coefficients. In general, deltas give more information about the trajectories of the MFCC coefficients over time. As we need fixed-size input to the proposed AFTViT architecture, we use 64 non-overlapping frames, 1 for each of the 64 Mel-bands, which gives a total array of 64 rows and 64 columns. The process for calculating 64 MFCCs involves the following steps: (1) Applying overlapping sliding windows to each heart sound segment. (2) Computing the Fast Fourier Transform (FFT) for each window. (3) Applying a mel filter bank and calculating its energies. (4) Performing a Discrete Cosine Transform (DCT) on the logarithmic filter bank energies. Herein, we use a window size of 1024 with no overlap. Consequently, three channels—MFCCs, along with their corresponding delta (Δ) and double-delta ($\Delta^{2}$) features—serve as the image-based input for the proposed architecture.

### 2.2. Two-Stream Vision Transformers Framework

The accuracy and complexity of the Vision Transformer (ViT) are influenced by the granularity of the chosen patch size. When using a finer patch size, the ViT tends to deliver improved performance. However, this comes at the expense of increased floating-point operations (FLOPs) and higher memory usage. For instance, a ViT with a patch size of 16 achieves a 6% improvement over one with a patch size of 32, but it requires four times more FLOPs. Taking into account this trade-off, our proposed method seeks to harness the advantages of various patch size.

To achieve this, the proposed AFTViT is structured such that each branch processes input patches at distinct scales. The ViT is capable of capturing high-level semantic information across various feature maps through the use of the self-attention mechanism within its transformer encoder. Specifically, the proposed architecture incorporates two branches: (1) S-Branch, a smaller branch functioning at a finer-grained patch size $P_{s}$, and (2) L-Branch, a larger branch utilizing a coarse-grained patch size $P_{l}$. In addition, we introduce a novel attention-driven fusion block designed to identify and combine the most discriminative features, merging the features from two streams into a single feature map, and finally, the deployment of a fully-connected layer is facilitated as a classifier head for the diagnosis of heart conditions, categorizing them as either diseased or healthy.

The proposed AFTViT framework can be mathematically represented as a quadruple $Q=(B_{s},B_{l},A,K)$. In this configuration, $B_{s}$ and $B_{l}$ represent the ViT encoder networks for two different sizes of feature patches, small and large, respectively. *A* denotes the attention-based fusion network, and *K* serves as the final classification component. The $I_{s}$ and $I_{l}$ represent the input feature maps that are generated through the preprocessing stage, as explained in Section 2.1. The features $f_{s}$ and $f_{l}$ are obtained from the ViT network having corresponding small-sized patches and large-sized patches, given by

(1) $$f_{s}=B_{s}\left(I_{s}\right)$$

(2) $$f_{l}=B_{l}\left(I_{l}\right)$$

These obtained feature maps are further fed to the attention-based fusion module *A* that combines the features into an integrated feature set $f_{A}$:

(3) $$f_{A}=A\left(f_{s},f_{l}\right)$$

Finally, the predicted identity label $\hat{l}$ is obtained using the classifier head *K*:

(4) $$\hat{l}=K\left(f_{A}\right)$$

The classifier head *K* is structured with three fully connected layers. The first two layers, each comprising 64 neurons, utilize the ReLU activation function. The final layer, sized according to the number of target classes, applies a softmax activation to yield classification results. The subsequent sections present a comprehensive overview of the components constituting the proposed AFTViT framework, beginning with an explanation of patch embedding and positional encoding. This is followed by a discussion on the fundamentals of self-attention and transformer encoder.

#### 2.2.1. Patch Embedding and Positional Encoding

**Figure 3:**
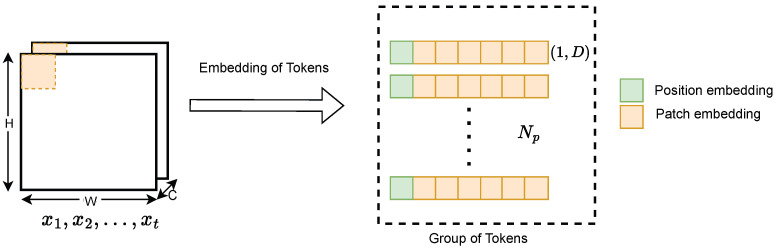
Patch and position embedding.

Our approach employs two separate ViT branches to independently analyze features from the feature map. This ViT framework is divided into two primary components, position and patch embedding (PPE) and the transformer encoder, as illustrated in Figure 1. Generally, the transformer model is designed to handle 1D sequences as input. Hence, converting image-based feature maps into one-dimensional representations becomes essential for processing. To achieve this, the input image *X*, with dimensions H×W×C, is divided into *N* equal-sized square patches, represented as $x_{1}^{p},x_{2}^{p},\ldots ,x_{N}^{p}$. Each patch $x_{i}^{p}$ belonging to $R^{H\times W\times C}$, is partitioned into a sequence of 2D patches, represented in $R^{N_{p}\times \left(P^{2}\cdot C\right)}$ space, as shown in Figure 3. In this representation, (H,W) represents the map’s resolution, *C* indicates the channel quantity, *P* represents the patch’s resolution, and $N_{p}$ equates to the count of patches, calculated as $\left(H\times W\right)/P^{2}$. The patches are linearly projected to a fixed dimension *D* within the encoder.

After the patch embedding process, positional embedding is introduced to encode spatial information for each patch in the sequence. This is achieved using a 1D approach, where patches are arranged sequentially from left to right and top to bottom. Additionally, a learnable class token, denoted as $x_{\mathrm{class}}$ and sized 1×D, is added at the beginning of the sequence to represent the class label. In the proposed AFTViT architecture, this token is denoted by ‘*’ to indicate its learnable nature. Positional encoding is crucial because, without it, the transformer would not distinguish between different images if the patch positions were altered. The result of combining patch and positional embeddings is given by

(5) $$Z_{0}=\left[x_{\mathrm{class}};x_{p}^{1}E;x_{p}^{2}E;\cdots ;x_{p}^{N}E\right]+E_{\mathrm{pos}}$$

Here, $x_{\mathrm{class}}$ represents the learnable class token; $x_{p}^{1};x_{p}^{2};\ldots ;x_{p}^{N}$ are the flattened patches, each of size 1 × $P^{2}C$; *E* is a trainable embedding matrix of size $P^{2}C\times D$; and $E_{\mathrm{pos}}$ consists of N+1 positional embedding vectors, each of size 1 × D. The variable $Z_{0}$ stands for the first layer input of the transformer encoder architecture.

As mentioned in Section 2.1, after the feature selection, the input heart sound signal is transformed into a 3-channel feature map, $I\in R^{64\times 64\times 3}$. As shown in Figure 1, our proposed architecture takes two stream input features. Specifically, for the small patch stream, input *I* is divided into patches of size 4×4, resulting in 256 patches (16×16 grid), each patch with depth 3. These patches are then projected into a 64-dimensional embedding space, whereas, for the large patch stream, using 8×8 patches results in 64 patches (8×8 grid), with the same depth, transformed similarly into 64-dimensional embeddings. After this, for the post-patch extraction process, each set of embeddings from the small and large patches is concatenated with their respective learnable classification tokens, $t_{small}$ and $t_{large}$, tailored to the projection dimension, making them $\in R^{1\times 64}$.

For each stream, patches are extracted and embedded separately based on their designated sizes, small and large, followed by the addition of a learnable classification token *t* to each set, formulated as

(6) $$f_{I_{small}}^{\prime }=\left[t_{small},f_{I_{small}}\right]$$

(7) $$f_{I_{large}}^{\prime }=\left[t_{large},f_{I_{large}}\right]$$

Positional knowledge for each patch is preserved by incorporating a learnable position matrix *P*, which embeds the location information. The dimensions of the position matrices for the small patch stream, $P_{\mathrm{small}}$, and the large patch stream, $P_{\mathrm{large}}$, are 257×64 and 65×64, respectively. The derived feature maps, $f_{P_{\mathrm{small}}}$$\in R^{257\times 64}$ and $f_{P_{\mathrm{large}}}$$\in R^{65\times 64}$, incorporate positional embeddings and serve as inputs to the transformer encoder, as given by

(8) $$f_{P_{small}}=P_{small}+f_{I_{small}}^{\prime }$$

(9) $$f_{P_{large}}=P_{large}+f_{I_{large}}^{\prime }$$

#### 2.2.2. Understanding Self-Attention

**Figure 4:**
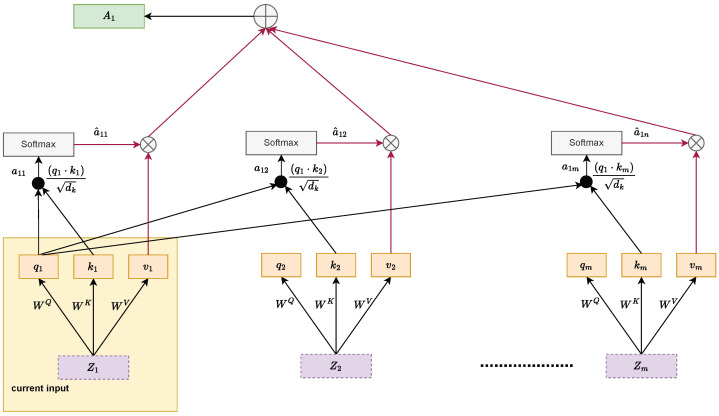
Computation of the self-attention score.

The authors of [39] introduced the attention mechanism for neural turning machines. Self-attention is a method where inputs interact with one another to create a meaningful representation of the input sequence using calculated attention values. This mechanism determines the relevance of all other inputs relative to a specific input. The process of self-attention is illustrated in Figure 4. This figure depicts the steps involved in calculating the self-attention score for an input sequence. Assume there are *m* input sequences $Z_{1},Z_{2},\ldots ,Z_{m}$, each with dimensions $1\times d_{f}$, where $d_{f}$ represents the embedding size. The initial step involves deriving the query ($q_{i}$), key ($k_{i}$) and value ($v_{i}$) vectors, where i = 1,2, …, m. This is achieved through the matrix multiplication of the input sequences with the trainable weight matrices $W^{Q}$, $W^{K}$, and $W^{V}$. The dimensions of these matrices are $d_{f}\times d_{q}$, $d_{f}\times d_{k}$, and $d_{f}\times d_{v}$, respectively. As a result of this transformation, the query, key, and value vectors acquire dimensions $1\times d_{q}$, $1\times d_{k}$, and $1\times d_{v}$, respectively. Since all input sequences are characterized by the same embedding size $d_{f}$, the dimensions of the query, key, and value vectors remain consistent, satisfying the condition $d_{q}=d_{k}=d_{v}$.

The process of computing the attention scores for a given input $Z_{1}$ begins by calculating the dot product between its query vector ($q_{1}$) and the key vectors ($k_{i}$), where i=1,2,…,m, corresponding to all inputs. These dot products are scaled by dividing them by the square root of the dimensionality of the key vectors ($\sqrt{d_{k}}$), resulting in intermediate values denoted as $a_{11},a_{12},\ldots ,a_{1m}$. The purpose of this scaling is to prevent the dot product values from becoming excessively large, which could hinder the effectiveness of the softmax function applied in the subsequent step. This approach is referred to as scaled dot product attention and serves as a key mechanism for stabilizing the computation. When the dimension $d_{k}$ is small, the scaling has minimal impact, as the raw dot products remain within a reasonable range. However, with larger $d_{k}$, the unscaled dot products can attain disproportionately high values, causing the softmax function to operate in regions with small gradients, which could result in optimization challenges such as vanishing gradients. Scaling by $\sqrt{d_{k}}$ addresses this issue effectively, ensuring the numerical stability of the attention mechanism. Once scaled, the dot product outputs are passed through the softmax function, which normalizes them to yield attention weights ${\hat{a}}_{11},{\hat{a}}_{12},\ldots ,{\hat{a}}_{1m}$.

(10) $${\hat{a}}_{1i}=\mathrm{softmax}\left(a_{1i}\right)=\mathrm{softmax}\left(\frac{q_{1}\cdot k_{i}}{\sqrt{d_{k}}}\right)$$

These weights are then utilized to compute the final attention vector $A_{1}$ by performing a weighted summation of the value vectors $v_{1},v_{2},\ldots ,v_{m}$. The resulting vector $A_{1}$ has a dimensionality of $1\times d_{v}$. Considering that *K* and *V* denote the key and value matrices, which are derived from the respective key and value vectors of all inputs, the self-attention score ($A_{1}$) for $Z_{1}$ can be mathematically expressed as follows:

(11) $$A_{1}=\mathrm{Attention}\left(q_{1},K,V\right)=\sum_{i=1}^{m}{\hat{a}}_{1i}v_{i}$$

where, $q_{1}$ represents the embedding query vector for input $Z_{1}$.

**Figure 5:**
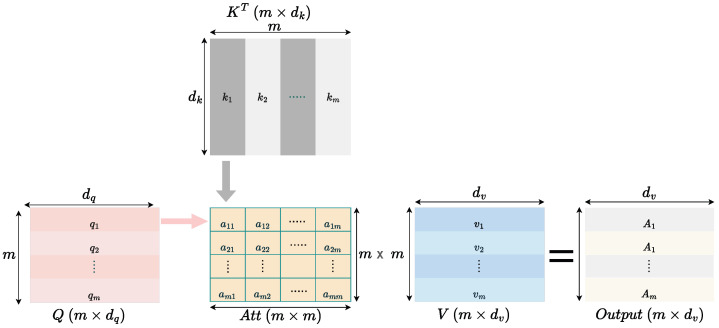
The self-attention mechanism represented through matrix multiplications.

This self-attention mechanism can also be interpreted using matrix operations, as shown in Figure 5. Let the input matrix *Z* be composed of *m* input embedding vectors $Z_{1},Z_{2},\ldots ,Z_{m}$, each of size $1\times d_{f}$, resulting in a matrix of size $m\times d_{f}$. The query (*Q*), key (*K*), and value (*V*) matrices are generated by multiplying *Z* by the corresponding trainable weight matrices $W^{Q}$, $W^{K}$, and $W^{V}$. The dimensions of *Q*, *K*, and *V* are $m\times d_{q}$, $m\times d_{k}$, and $m\times d_{v}$, respectively. The attention scores are calculated by multiplying *Q* by the transpose of *K*, resulting in an attention score matrix of size m×m. These scores are scaled by $\frac{1}{\sqrt{d_{k}}}$ to stabilize the computations and normalized using the softmax function, yielding the attention weights. Finally, these weights are applied to *V*, producing the self-attention output matrix with dimensions $m\times d_{v}$. This process is mathematically represented as

(12) $$\mathrm{Attention}\left(Q,K,V\right)=\mathrm{softmax}\left(\frac{QK^{T}}{\sqrt{d_{k}}}\right)V$$

where *Q*, *K*, and *V* represent the query, key, and value matrices, respectively, and $d_{k}$ is the dimension of the key vectors.

#### 2.2.3. Transformer Encoder

**Figure 6:**
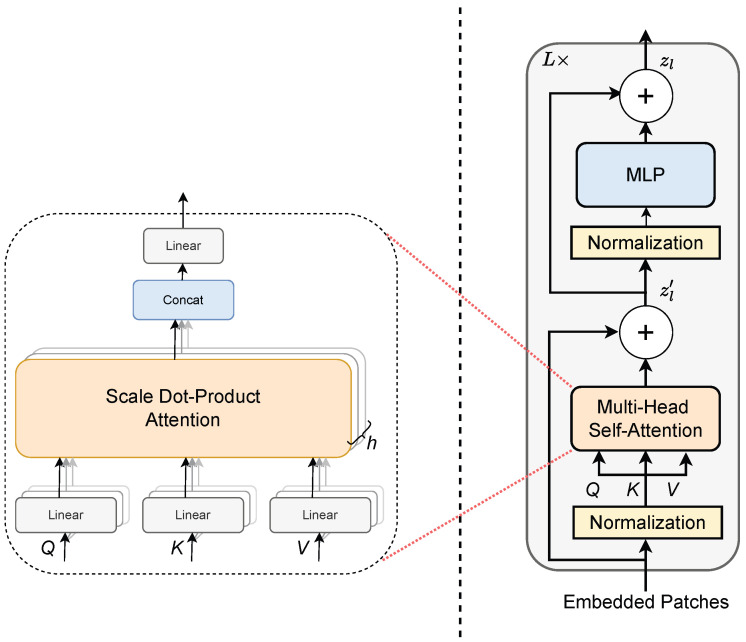
The left panel illustrates the multi-head self-attention (MSA) mechanism, highlighting its parallel attention heads, while the right panel depicts the structural details of the transformer encoder.

The multi-head self-attention (MSA) mechanism extends standard self-attention (SA), as shown on the left side of Figure 6. It performs parallel computations across *h* independent heads. Each head focuses on a different part of the input, capturing diverse information. Scaled dot-product attention is used in each head. Self-attention can be calculated independently for each head using Equation (12). The query, key, and value matrices are computed using learnable weight matrices ($W^{Q}$, $W^{K}$, $W^{V}$). This design allows the model to handle complex sequence data effectively and extract detailed features. The MSA mechanism combines the outputs from multiple self-attention heads to produce a final output matrix of size $\left(N+1\right)\times d_{s}\cdot h$, where N+1 includes the input sequence length and a learnable class token. Here, $d_{s}$ corresponds to the value vector dimension and *h* denotes the number of attention heads. The combined output is then passed through a linear projection using the MSA weight matrix $U_{msa}$, with dimensions $h\cdot d_{s}\times d_{o}$, where $d_{o}$ is the output feature dimension. This results in the final MSA output matrix of size $\left(N+1\right)\times d_{o}$. The overall MSA computation for *h* heads is expressed as

(13) $$\mathrm{MSA}\left(Z\right)=\left[{\mathrm{SA}}_{1}\left(Z\right);{\mathrm{SA}}_{2}\left(Z\right);\cdots ;{\mathrm{SA}}_{h}\left(Z\right)\right]U_{msa}$$

where ${\mathrm{SA}}_{i}\left(Z\right)$ is the self-attention output from the *i*th head.

As shown on the right side of Figure 6, the transformer encoder is composed of an MSA module and an MLP module, with Layer Normalization (LN) applied prior to each component. The MLP block is composed of two-layer fully connected layers. Residual connections are employed after every block to facilitate gradient flow and stabilize training. In a transformer encoder, *L* denotes the number of encoder blocks in the model. In our design, both branches are configured with an equal number of encoder blocks, where *L* is set to 5. The outputs of the MSA block and the Transformer encoder are represented as $Z_{l}^{\prime }$ and $Z_{l}$, respectively, and can be mathematically expressed by Equations (14) and (15).

(14) $$Z_{l}^{\prime }=\mathrm{MSA}\left(\mathrm{LN}\left(Z_{l-1}\right)\right)+Z_{l-1},\;l=1,\ldots ,L$$

(15) $$\begin{array}{cc} Z_{l} & =\mathrm{MLP}\left(\mathrm{LN}\left(Z_{l}^{\prime }\right)\right)+Z_{l}^{\prime },\;l=1,\ldots ,L \end{array}$$

### 2.3. Attention Fusion Block for Feature Integration

**Figure 7:**
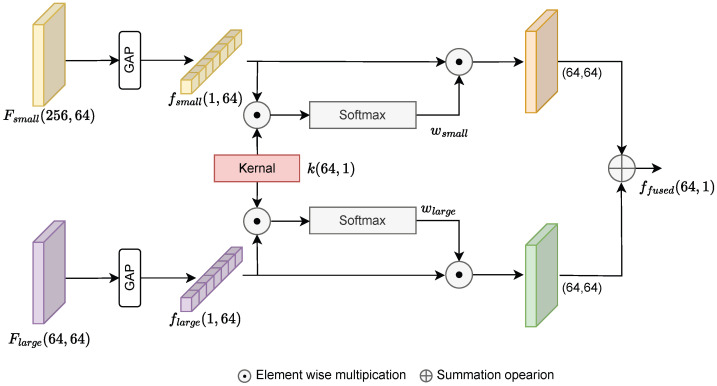
Proposed attention fusion block.

As shown in Figure 7, we design the Attention Fusion Block (AFB) to integrate features from two input streams, termed explicitly as the small and large streams, to greatly increase the effectiveness of the model. Unlike D-Mixer [40], which integrates global and local dependencies within each block of a single-branch network, our method employs two ViT encoders at different patch scales and integrates their outputs at the feature level through the proposed attention fusion block. The process begins with two sets of input features maps input feature $f_{\mathrm{small}}\in R^{256\times 64}$ and $f_{\mathrm{large}}\in R^{64\times 64}$, from the small and large branch, respectively. An essential first step is to align these feature dimensions into $f_{\mathrm{small}}\in R^{1\times 64}$, and $f_{\mathrm{large}}\in R^{1\times 64}$ using a global average pooling (GAP) operation, which facilitates coherent processing. The core component of our attention framework is the kernel $k\in R^{64\times 1}$, which plays a crucial role during training. In the proposed implementation, the kernel *k* is a trainable parameter vector, initialized using Glorot uniform initialization and updated jointly with the rest of the network via backpropagation. This kernel facilitates the calculation of attention scores, a key component in determining the relevance of features from each input stream.

In our approach, we compute the attention scores for each respective branch as follows:

(16) $$s_{\mathrm{small}}=f_{\mathrm{small}}⊙k$$

(17) $$s_{\mathrm{large}}=f_{\mathrm{large}}⊙k$$

where ⊙ denotes the element wise multiplication. These raw attention scores are then normalized via the softmax function to derive the attention weights, ensuring a balanced contribution across features:

(18) $$w_{\mathrm{small}}=\mathrm{softmax}\left(s_{\mathrm{small}}\right)$$

(19) $$w_{\mathrm{large}}=\mathrm{softmax}\left(s_{\mathrm{large}}\right)$$

After the attention weights are computed, the next step is their application to the respective feature sets via element-wise multiplication. The weighted features are aggregated to form a single, fused feature representation, $f_{\mathrm{fused}}$:

(20) $$f_{\mathrm{fused}}=\sum \left(w_{\mathrm{small}}⊙f_{\mathrm{small}}\right)+\sum \left(w_{\mathrm{large}}⊙f_{\mathrm{large}}\right)$$

where ∑ denotes summation over the feature dimensions. This results in a cohesive feature map that encapsulates information from both input streams. The combined information, $f_{\mathrm{fused}}$, is further fed to the classifier. The attention fusion block effectively integrates features from two streams by leveraging attention mechanisms. This approach ensures that the most relevant information is retained, improving the model’s overall performance.

## 3. Results and Discussion

### 3.1. Experimental Setup and Evaluation Metrics

To evaluate our proposed method, we utilize the PhysioNet2016 [41] and PhysioNet2022 [42] datasets. The PhysioNet2016 dataset comprises eight independent subsets, collected by seven different research teams. These subsets exhibit significant variability in terms of collection environments, acquisition devices, recording locations, and patient types. All heart sound recordings were sampled at a frequency of 2000 Hz, with durations ranging from 5 s to just over 120 s. These phonocardiogram (PCG) recordings were collected globally, encompassing both clinical and nonclinical environments. The dataset categorizes the recordings into two classes: normal and abnormal. Abnormal recordings correspond to patients with confirmed CVDs, while normal recordings were collected from healthy individuals. Although the dataset includes patients with various heart conditions, its primary focus is on identifying abnormalities rather than classifying specific heart diseases. The proposed architecture is further assessed for its performance using the PhysioNet2022 dataset, the most comprehensive public heart sound dataset to date. Physionet2022 contains three distinct categories, namely, present, absent, and unknown, instead of the conventional binary classification. The “present” label signifies the detection of a heart murmur, while “absent” indicates that no murmur is found. The “unknown” category is assigned to cases where the data is unclear or inconclusive. Including this category is essential in medical classification as it prevents misclassification and ensures that uncertain cases are reviewed by medical professionals, ultimately improving patient safety. The dataset comprises 5272 heart sound recordings collected from the primary auscultation points of 1568 patients. Recordings were taken from four key auscultation areas: aortic area, pulmonary area, tricuspid area, and mitral area.

The selection of patch size is a key factor influencing both the performance and stability of the designed architecture. To address the challenges associated with determining the optimal patch size ratio, we considered several options, namely, 2 × 2, 4 × 4, 8 × 8, and 16 × 16, based on our image-based feature representation. The choice of 4 × 4 and 8 × 8 patches was made after careful consideration of the nature of the input representation and the demands of the fusion stage. For the 64 × 64 image-based feature map, a 4 × 4 patching scheme results in 256 tokens, allowing the small-patch branch to capture fine temporal and spectral details, which are crucial for identifying murmurs and other localized heart sound events. In contrast, an 8 × 8 patching scheme generates 64 tokens, reducing the computational burden while still preserving the broader context of the signal. Thus, we selected the two branches to complement each other: one focuses on fine details, while the other captures more global patterns.

We also explored alternative patch configurations. Smaller patches, such as 2 × 2, lead to the generation of up to 1024 tokens, which significantly increases the computational load. On the other hand, larger patches, such as 16 × 16, produce only 16 tokens, resulting in overly coarse representations that lack the fine-grained local information required for effective classification. Additionally, when the difference in patch sizes is too large, the outputs of the two branches become unbalanced. During fusion, the branch with the larger number of tokens tends to dominate, reducing the contribution of the other branch. By selecting 4 × 4 and 8 × 8 patches, we obtained a balanced ratio where neither branch overwhelms the other, leading to stable and effective fusion. For these reasons, we adopted the 4 × 4 vs. 8 × 8 configuration in this work.

The experiments were conducted on an Nvidia RTX3090 GPU using tensorflow and keras as the backend frameworks. The binary cross-entropy and categorical cross-entropy functions are employed to compute the loss for the PhysioNet2016 and PhysioNet2022 datasets, respectively. In both scenarios, an adam optimizer is applied with an initial learning rate of 0.008 and no learning rate scheduling. The network is trained for up to 400 epochs using an early stopping strategy. If validation performance does not improve for 15 consecutive epochs, training stops, and the best weights from the epoch with the highest validation performance are restored. A batch size of 32 is used. The dataset is partitioned into training, validation, and test subsets. A 10-fold cross-validation is performed only on the 90% data used for training and validation, while the 10% test set is kept separate and reserved solely for final evaluation. Additionally, the splitting was carried out on a subject-wise basis to ensure that no subject appeared in more than one subset within the same fold, thereby eliminating any chance of data leakage. The training set is utilized to update the network’s parameters for task optimization, whereas the testing set is employed to evaluate the performance of the trained model and measure classification accuracy based on the output results. The proposed model’s performance is evaluated based on the following metrics:

$$\mathrm{Sensitivity}=\frac{TP}{TP+FN}$$

$$\mathrm{Specificity}=\frac{TN}{TN+FP}$$

$$F1\;\mathrm{Score}=\frac{2\times TP}{2\times TP+FP+FN}$$

$$\mathrm{Accuracy}=\frac{TP+TN}{TP+FP+TN+FN}$$

$$\mathrm{Score}=\frac{Sensitivity+Specificity}{2}$$

where, TP, TN, FP, and FN denote the counts of true positives, true negatives, false positives, and false negatives, respectively.

### 3.2. Comparison of Fusion Methods

Experiments are carried out to verify the importance of the attention fusion mechanism in the proposed network. As explained in Section 2.3, the output feature maps of the respective ViT branch are fed to the proposed attention block. The attention mechanism effectively captures the interrelations among feature maps, thereby enhancing the model’s representational capability. Furthermore, various fusion mechanisms, namely, average, min, max, and concatenation, can be adopted in the proposed two-stream framework. We applied global average pooling to the feature map output from the ViT branch to achieve uniform dimensionality for fusion operations.

**Figure 8:**
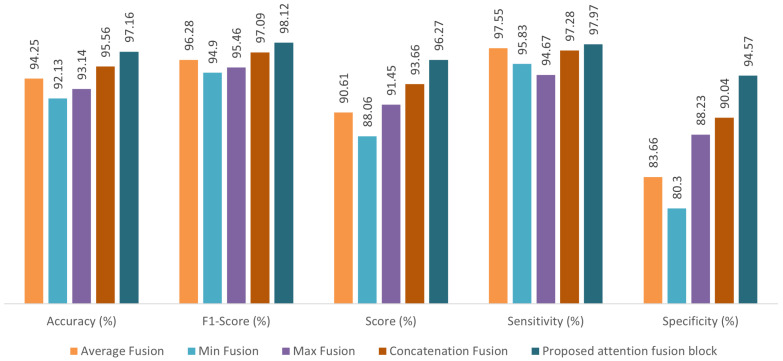
Performance comparison of fusion methods on the PhysioNet2016 heart sound dataset.

**Figure 9:**
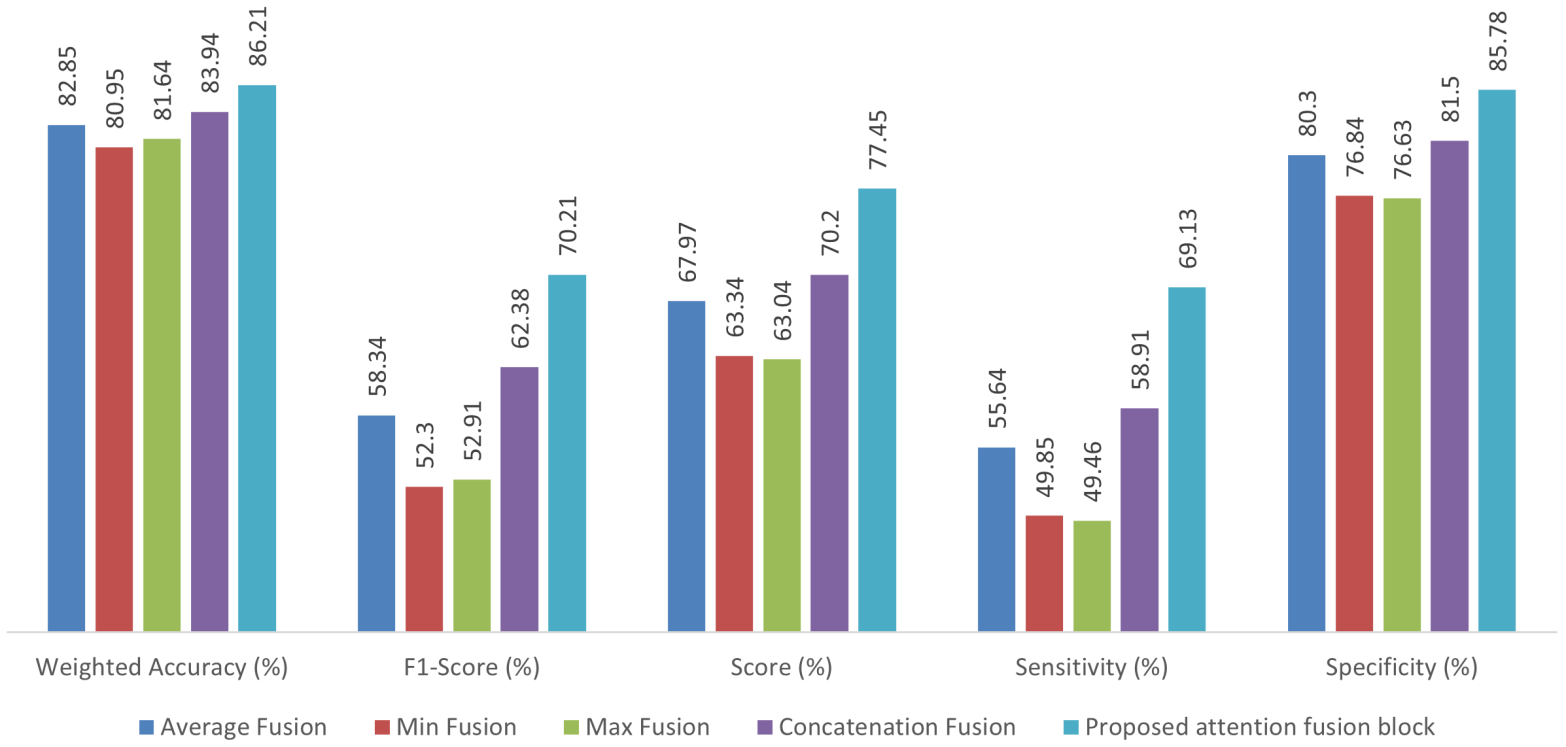
Performance comparison of fusion methods on the PhysioNet2022 heart sound dataset.

Figure 8 and Figure 9 show the performance parameters of different fusion configurations for the PhysioNet2016 and PhysioNet2022 datasets, respectively. Since the PhysioNet2022 dataset contains three classes of heart sounds, we chose a weighted accuracy to ensure a balanced evaluation of the model’s performance. Weighted accuracy is important in multi-class scenarios where certain classes have significantly fewer samples than others. Instead of equally considering all classes, weighted accuracy assigns class-specific weights to ensure a more representative performance evaluation. This approach minimizes bias toward majority classes and enhances the reliability of model performance assessment. It is noted that for the PhysioNet2016 dataset, the proposed attention fusion block improves the classification accuracy by 2.91%, 5.03%, 4.02% and 1.60% compared to average, min, max, and concatenation fusion methods, respectively. The corresponding values of weighted accuracy for the PhysioNet2022 dataset are 3.36%, 5.26%, 4.57%, and 2.27%, respectively. A similar trend is observed for other performance parameters such as score, specificity, F1-score and sensitivity. The results of this experiment indicate that the proposed attention module improves the overall performance of the architecture compared to other fusion techniques. It can also be observed that the concatenation operation yields better results compared to Min, Max and average fusion since it preserves the feature map by combining it, thereby providing discriminative features. In general, the proposed attention fusion block configuration outperforms the others due to the benefits provided by the attention fusion. Hence, we employ this method in the proposed two-stream framework.

### 3.3. Performance Against Other CNN Approaches

This subsection compares the proposed architecture’s performance with that of other CNN-based approaches on the two datasets. We consider popular models, namely VGG19 [43], ResNet50 [44], Inception-v3 [45], and Xception [46]. For a fair comparison, the classifier configurations are kept the same for all CNN approaches. In addition, the input dimensions of 224×224 for these models are maintained after performing the resize operation. Extensive comparisons are conducted using different evaluation metrics and the number of training parameters. The results obtained from the experiments on the PhysioNet2016 and PhysioNet2022 datasets are presented in Table 1 and Table 2, respectively. It can be observed that the proposed AFTViT outperforms all baseline models across both datasets, demonstrating its effectiveness in HSC. On the PhysioNet2016 dataset, as shown in Table 1, the proposed AFTViT achieves an accuracy of 97.16%, significantly higher than the best-performing baseline, Xception, which records 93.36%. Furthermore, the proposed AFTViT achieves, F1-score, score, sensitivity and specificity of 98.12%, 96.27%, 97.97%, and 94.57%, respectively, which demonstrate significant improvement over other CNN methods. A similar trend is observed on the PhysioNet2022 dataset, where the proposed AFTViT continues to outperform other CNN methods. AFTViT achieves a weighted accuracy of 86.21%, which is a 3.79% improvement from the best-performing Inception-v3 model. Additionally, AFTViT achieves the highest F1-score (70.21%), score (69.13%), sensitivity (85.78%), and specificity (77.45%), indicating its robust performance. Notably, AFTViT achieves these results while requiring only 0.86 million parameters, making it far more efficient than other baseline models. The results clearly indicate that the proposed AFTViT not only achieves superior performance across diverse datasets but also attains significant computational efficiency.

### 3.4. Performance Against State-of-the-Art Approaches

This part explores the performance of the proposed AFTViT network against state-of-the-art CNN-based architectures for HSC. Table 3 presents a comprehensive analysis of the classification accuracies and the number of training parameters for all approaches. The findings demonstrate that the proposed AFTViT network achieves a significant improvement in accuracy, attaining 97.16%, while maintaining an exceptionally low parameter count of 0.86 M. The ResNet152 model, which employs MFCC and Fbank features in an early fusion two-branch architecture, achieves an accuracy of 86.20% but requires 138.85 M parameters. Despite utilizing two types of features, its performance is limited due to early fusion. Similarly, PANNs, a widely used model for audio classification, achieves 89.7% accuracy with a two-branch CNN network. However, this model requires 80.7 M parameters, which is approximately 80 times the parameter count of the proposed model. These observations highlight the computational inefficiency of these approaches compared to the proposed AFTViT model. AlexNet, VGG16, and Inception-v3, all of which are single-branch networks utilizing chaogram features derived from PCG signals, achieve classification accuracies of 89.68%, 90.05%, and 90.36%, respectively. These models, however, require parameter counts of 61 M, 138 M, and 23.9 M, respectively. The proposed AFTViT-based approach not only surpasses existing methods in terms of classification accuracy but also achieves a notable reduction in computational complexity. It significantly outperforms other methods, such as ResNet152 and PANNs, which demand considerably higher computational resources. Among these methods, Inception-v3 emerges as the closest competitor to the proposed model, demonstrating performance that is approximately 7% lower in accuracy compared to the proposed approach and necessitates 30 times more parameters. Despite their strong performance, these methods fall short of the proposed AFTViT model, which outperforms them by a substantial margin. These results establish the proposed AFTViT architecture as a robust and efficient solution for HSC.

To further evaluate the performance of the proposed model, experiments were conducted on a secondary dataset. Table 4 highlights the weighted accuracy and required number of parameters for the proposed approach in comparison to various CNN-based state-of-the-art models on the PhysioNet2022 dataset. The findings indicate a significant advancement in accuracy by the proposed approach, increasing from 75.6% to 86.21%, representing a 10.61% enhancement. Notably, the proposed AFTViT method achieves the highest weighted accuracy of 86.21%. Most of these methods rely on pre-trained models trained on either audio or image datasets. In [9], ResNet50, a single-branch network, achieves a weighted accuracy of 75.6% with 14.7 million parameters. The HMT model [10], employing a three-branch ResNet architecture with late fusion, shows inferior performance despite utilizing late-fusion techniques. This limitation arises due to the use of identical feature inputs and the lack of multi-resolution features. Furthermore, this method significantly increases computational demands, requiring 204 million parameters. Wave2Vec, a widely adopted audio model, achieves a weighted accuracy of 80%, which is comparable to the proposed approach. However, this model’s parameter count is significantly higher than that of the proposed model, highlighting its computational inefficiency. The proposed approach effectively utilizes a ViT-based transformer architecture to extract features at varying resolutions and integrates a feature fusion block employing a fusion strategy. This enables the selection of more relevant and noise-resilient features, thereby enhancing overall performance.

Although this study utilizes a two-branch network, it is important to highlight that the proposed Attention fusion block integrates single-domain features across multiple resolutions. This model offers flexibility, allowing for the seamless integration of additional features at varying resolutions through optimized ViT-based feature extractors. However, such extensions would increase the framework’s overall size and could potentially restrict its practicality in real-world scenarios. Nonetheless, the results clearly demonstrate that leveraging features at two resolution levels achieves excellent performance. The proposed model exhibits consistent and reliable performance across both datasets, utilizing minimal parameters. These outcomes validate the robustness and efficacy of the suggested dual-stream architecture. Adopting this model holds promise for substantial advancements in public health, demonstrating its potential for a widespread positive impact.

## 4. Conclusions

In this paper, we have designed a novel two-stream ViT-based architecture, enhanced with an attention fusion block for HSC, called AFTViT. The architecture employs a feature-level attention mechanism designed to explore diversified feature representations by capturing cross-relations across different scales. Specifically, the ViT encoder processes inputs of varying resolutions, with the small branch focusing on fine-grained features from smaller patches and the large branch leveraging larger patches to capture broader contextual information. To achieve effective representation, MFCCs and their delta features were extracted from raw heart sound signals, providing a robust feature representation. These features are separately processed through ViT feature extractors, and the outputs are integrated using an advanced attention fusion module. The attention fusion block subsequently integrates these representations at the feature level, yielding highly discriminative features for classification. AFTViT’s modular design demonstrates its scalability, allowing seamless incorporation of additional feature spaces by including new branches. Comprehensive experiments illustrate that the attention module outperforms conventional fusion techniques. Moreover, AFTViT outperforms other CNN models, underscoring its efficacy. The integration of complementary feature representations at different scales within AFTViT results in substantial improvements in classification performance. The proposed method achieves state-of-the-art results on publicly available datasets, PhysioNet2016 and PhysioNet2022, highlighting its potential for advancing automated HSC.

## Figures and Tables

**Table 1:** Comparison of CNN architectures on the PhysioNet2016 heart sound dataset.

Baseline Network	Accuracy (%)	F1-Score (%)	Score (%)	Sensitivity (%)	Specificity (%)	Parameters (M)
VGG19	89.97	93.62	83.95	95.44	72.46	20.06
ResNet50	91.08	94.18	87.13	94.66	79.61	23.72
Inception-v3	91.41	94.38	87.92	94.59	81.25	21.94
Xception	93.36	95.62	90.78	95.71	85.86	20.99
Proposed AFTViT	97.16	98.12	96.27	97.97	94.57	0.86

**Table 2:** Comparison of CNN architectures on the PhysioNet2022 heart sound dataset.

Model	Weighted Accuracy (%)	F1-Score (%)	Score (%)	Sensitivity (%)	Specificity (%)	Parameters (M)
VGG19	77.92	41.29	56.19	40.55	71.84	20.06
ResNet50	79.17	45.39	58.42	43.44	73.40	23.72
Inception-v3	82.42	57.49	66.91	54.73	79.08	21.94
Xception	81.24	52.11	63.11	49.25	76.97	20.99
Proposed AFTViT	86.21	70.21	69.13	85.78	77.45	0.86

**Table 3:** Performance comparison with state-of-the-art CNN-based approaches on the PhysioNet2016 dataset.

Network	Features	Configuration	Accuracy	Number of Parameters
ResNet152 [20]—2022	Fbank & MFCCs	early fusion, two branch	86.20	138.85 M
AlexNet [10]—2022	chaogram features	single branch	89.68	61 M
PANNs [24]	raw signal	feature-level fusion, two branch	89.7	80.7 M
VGG16 [10]—2022	chaogram features	single branch	90.05	138 M
Inception-v3 [10]—2022	chaogram features	single branch	90.36	23.9M
Proposed AFTViT	MFCCs	feature-level fusion, two branch	97.16	0.86 M

**Table 4:** Performance comparison with state-of-the-art CNN-based approaches on the PhysioNet2022 dataset.

Network	Features	Configuration	Weighted Accuracy	Number of Parameters
ResNet50 [14]	raw signal	single branch	75.6	14.7 M
HMT [21]	log-mel spectrogram	late fusion, three branch	78.3	204 M
wav2vec model [15]	raw signal	single branch	80	317 M
Proposed AFTViT	MFCCs	feature-level fusion, two branch	86.21	0.86 M

## Data Availability

The data used in this study are publicly available from the respective websites: https://physionet.org/content/challenge-2016/1.0.0/ (accessed on 24 July 2025) and https://moody-challenge.physionet.org/2022/ (accessed on 24 July 2025).
